# Appendix Anchoring Approach: A novel method to prevent appendicitis post-appendiceal endoscopic submucosal dissection

**DOI:** 10.1055/a-2678-9799

**Published:** 2025-08-22

**Authors:** Yohei Koyama, Miwako Arima, Terumitsu Anai, Takuto Hida, Yoriyuki Takamori, Akihiko Tsuchiya, Kou Nishikawa

**Affiliations:** 1 13959 Department of Gastroenterology and Hepatology, Ageo Central General Hospital, Ageo, Japan

Endoscopic submucosal dissection (ESD) of cecal tumors extending into the appendiceal orifice is technically challenging and associated with a high risk of perforation and post-ESD appendicitis 1 2 . Here, we report a novel technique for preventing post-ESD appendicitis: the appendix anchoring approach ( Video 1 ).

Appendix anchoring approach: A novel technique for the prevention of appendicitis post-appendiceal endoscopic submucosal dissection.Video 1

A 95-year-old man with no history of appendectomy presented with a 60-mm laterally spreading tumor (LST) in the cecum that completely covered and extended into the appendiceal orifice ( Fig. 1 ). The LST was resected by ESD using the water pressure and countertraction method 3 4 5 . After circumferential incision, submucosal dissection around the appendix was performed to the extent possible, and the appendix was exposed beneath the lesion. Sufficient traction was achieved in the appendix when a traction clip was applied. The dissected appendiceal mucosa was inverted into the cecum by continuous traction, enabling the submucosal dissection to continue toward the tip of the appendix ( Fig. 2 ). However, a minor appendiceal mucosal tear occurred due to excessive tension. Through the tear, the appendiceal lumen was lined with non-neoplastic mucosa, leading to the decision to resect the appendix at that level. However, this raised concerns regarding the burial of the residual appendiceal mucosa, potentially increasing the risk of obstructive appendicitis ( Fig. 3 **a,b** ). Therefore, the distal appendix at the site of the mucosal tear was grasped with a clip, and only the semi-circumference of the appendix was anchored to the cecal wall to maintain luminal patency ( Fig. 3 **c,d** ). The lesion was resected en bloc by cutting immediately above the clip. The fixed appendiceal lumen remained patent and was expected to be the drainage lumen. No adverse events occurred intra- or postoperatively. The tumor was histologically diagnosed as an intramucosal adenocarcinoma and curative resection was achieved.

**Fig. 1:**
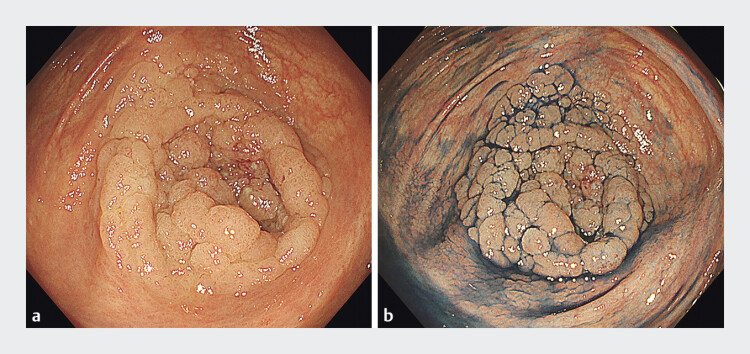
Endoscopic images of the tumor. **a** A 60-mm laterally spreading tumor in the inferior aspect of the cecum that completely covered and extended into the appendiceal orifice. **b** Indigo carmine chromoendoscopy image. The distal margin of the laterally spreading tumor within the appendix could not be visualized endoscopically.

**Fig. 2:**
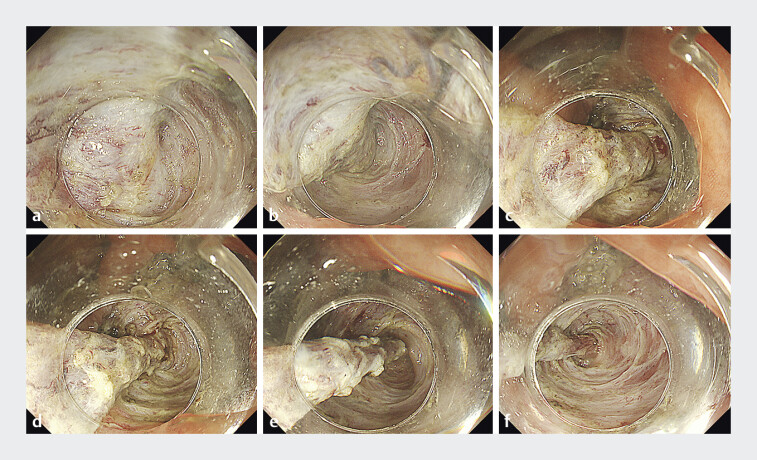
Process of submucosal dissection within the appendix. **a** – **f** Images showing the progression of submucosal dissection within the appendix. The dissected appendiceal mucosa was inverted into the cecum by continuous traction.

**Fig. 3:**
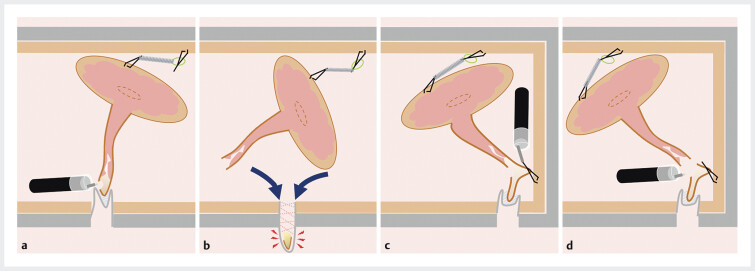
Schema of appendiceal resection at a level proximal to the tip. **a** Resection of the appendix at the level of the mucosal tear. **b** Resecting the appendix at the level of the mucosal tear could lead to the burial of the residual appendiceal mucosa, potentially increasing the risk of obstructive appendicitis. **c** The distal appendix at the site of the mucosal tear was grasped using a clip. To preserve luminal patency, only a semi-circumference of the appendix was anchored to the cecal wall. **d** En bloc resection by cutting just above the clip.

The appendix anchoring approach is a simple procedure that may prevent post-ESD appendicitis when the appendix is resected proximal to the tip.

Endoscopy_UCTN_Code_TTT_1AQ_2AD_3AD

## Notes

Endoscopy E-Videos https://eref.thieme.de/e-videos
*E-Videos* is an open access online section of the journal *Endoscopy* , reporting on interesting cases and new techniques in gastroenterological endoscopy. All papers include a high-quality video and are published with a Creative Commons CC-BY license. Endoscopy E-Videos qualify for HINARI discounts and waivers and eligibility is automatically checked during the submission process. We grant 100% waivers to articles whose corresponding authors are based in Group A countries and 50% waivers to those who are based in Group B countries as classified by Research4Life (see: https://www.research4life.org/access/eligibility/ ). This section has its own submission website at https://mc.manuscriptcentral.com/e-videos .
